# Effects of dietary *Chinese yam— Rehmannia glutinosa* ‘medicine pair’ on growth performance, body size trait, slaughtering performance, antioxidant capacity, and cecum microbiota of broilers

**DOI:** 10.3389/fnut.2026.1784952

**Published:** 2026-02-25

**Authors:** Jinliang Zhang, Yan Guo, Yanan Ding, Bingbing Ma, Zhiqiang Li, Huijun Zhang

**Affiliations:** Department of Animal Science, College of Biology and Food, Shangqiu Normal University, Shangqiu, Henan, China

**Keywords:** broiler, cecum microbiota, *Chinese yam*, medicine pair, *Rehmannia glutinosa*

## Abstract

**Objectives:**

*Chinese yam* and *Rehmannia glutinosa*, Henan province local medicinal herbs, have received attention owing to its positive nutritional and medicinal characteristics. The combination of *Chinese yam* and *Rehmannia glutinosa*, known as a ‘medicine pair’, has a long history of use. However, no published study has evaluated *Chinese yam*—*Rehmannia glutinosa* medicine pair (CYRG) as a dietary additive in broilers. This study aimed to evaluate the impact of dietary CYRG on growth performance, body measurements, slaughtering performance, antioxidant capacity and cecum microbiota of broilers.

**Methods:**

Total of 300 23-day-old Arbor Acres female broilers were randomly assigned to the 5 treatment groups (CON, 0.5% CYRG, 1% CYRG, 1.5% CYRG, and 2% CYRG) with 6 replications and 10 birds/replicate. In the CON group, broilers received the basal diet, and other 4 groups received the basal diet supplemented with 0.5, 1, 1.5, and 2% CYRG, respectively. The feeding trial lasted for 19 days, until to 42 days.

**Results:**

The results showed that 0.5% CYRG group had the highest of body weight gain, average daily gain, and average daily feed intake among the 5 groups. Supplementation CYRG significantly improved the slaughter percentage and semi-evisceration weight percentage of broilers (*P* < 0.05). Supplementation 0.5% CYRG and 1% CYRG significantly increased the level of anti-inflammatory cytokines (IL-4) and decreased the pro-inflammatory cytokines (IL-6) level (*P* < 0.05). Supplementation 1.5% CYRG improved the levels of serum immunoglobulins G (IgG) and IgM (*P* < 0.05). 0.5% CYRG significantly increased the levels of catalase (CAT), glutathione peroxidase (GSH-Px), and total antioxidant capacity (T- AOC), and decreased the levels of malondialdehyde (MDA) (*P* < 0.05). Microbiological analysis showed that CYRG supplementation increased the abundance of beneficial bacteria (Firmicutes) and decreased the quantity of harmful bacteria (Proteobacteria and Cyanobacteria). Correlation analysis indicated that *Lactobacillus salivarius* and *Lactobacillus_crispatus* were positively associated with serum IgA and IgG (*P*<0.05). And *Ruminococcaceae* were positively correlated with CAT and GSH-Px activities (*P*<0.05), and *Unclassified Clostridia ucG 014* was negatively correlated with MDA (*P*<0.05).

**Conclusion:**

Results showed that dietary CYRG improved the economic benefits of broilers by improving slaughter performance, physical and intestinal health. The 0.5% CYRG dose was recommended.

## 1 Introduction

*Chinese yam* is a very popular tuber crop cultivated as a functional food and traditional medicine in China (1–4). According to the ‘Compendium of Materia Medica’, *Chinese yam* can ‘strengthen the kidney qi, invigorate the spleen and stomach, stop diarrhea and dysentery, disperse phlegm and saliva, moisturize the skin’, and modern medical research has proved that *Chinese yam* contains polysaccharides, polyphenols, terpenoids, steroids, peptides, alkaloids, and essential oils and other bioactive ingredients (5, 6). Previous studies have shown that it has various pharmacological effects, for example, promoting growth performance (3), improving meat quality (7), enhancing antioxidant capacity (8) and immunity (9), and improving the intestinal microbial community (10) in animal. *Rehmannia glutinosa* was first recorded in Shennong’s Classic of Materia Medica and was classified as an ‘excellent’ herb. As a traditional Chinese herbal medicine, it mainly including iridoid glycosides, phenylpropanoid glycosides, saccharides, and unsaturated fatty acids active ingredients, and it possesses multiple effects, including anti-inflammation, anti-tumor, enhancing immunity, anti-aging and antioxidation (11). Yang et al. (12) found that 600 and 900 mg/kg *Radix rehmanniae* preparata polysaccharide improved broilers’ growth, gut physiology, and tibia ash content. Yu et al. (13) found that *Rehmannia glutinosa* polysaccharides enhanced intestine immunity by altering the gut microbiota composition in mice. Quan et al. (14) found that *Rehmannia glutinosa* water extract improved blood lipid levels, improved antioxidant capacity of kidney and liver function in diabetic nephropathy rats.

The ‘medicine pair’ of *Chinese yam* and raw *Rehmannia glutinosa*, in a ratio of 1:1 to 1:2, was described in the Ming Dynasty’s ‘Zhang Xichun’s Herbal Pairings’, ‘When these two herbs are combined, their efficacy is greatly enhanced’. However, up to now, no published study has evaluated *Chinese yam* - *Rehmannia glutinosa* ‘medicine pair’ (CYRG) as a dietary additive in animal. In this study, we prepared CYRG, evaluated the effects of the dietary supplementation of CYRG on the growth performance, body size trait, slaughtering performance, antioxidant capacity and cecum microbiota of broilers, and provided a basis for the scientifically application of CYRG.

## 2 Materials and methods

### 2.1 Animal care

All birds in this study feeding program were approved by the Animal Ethics Committee of Shangqiu Normal University (2023–1,109).

### 2.2 Preparation of medicine pair (CYRG)

*Chinese yam* and *Rehmannia glutinosa* were obtained from Wuzhi county, Jiaozuo city, Henan Province, China in 2024. At the time, all samples were dried and crushed. *Chinese yam* passed through an 80-mesh sieve, and *Rehmannia glutinosa* with a particle size pass 40 mesh. Then prepared the medicine pair (CYRG) by mixing *Chinese yam* powder and *Rehmannia glutinosa* powder in a 1:1 (m/m) ratio. Then, CYRG was stored in a cool, dry place until use.

### 2.3 Experimental diets and animal management

Three hundred healthy 23-day-old Arbor Acres female broilers with similar body weight were randomly placed into five groups with 6 replicates, with 10 broilers per replicate. In the control group (CON), broilers received the basal diet, and other treatment groups (0.5% CYRG, 1% CYRG, 1.5% CYRG, and 2% CYRG) received the basal diet supplemented with 0.5, 1, 1.5, and 2% CYRG, respectively. The feeding experiment last for 19 days, until the birds were 42 days old. The basic diet was formulated to reference to the National Research Council (1994) (15), and Table 1 shows the ingredients of the basal diet. Broilers were caged, and had free access to diet and water during the experiment. Birdhouse temperature was kept at 24 ± 2 °C and under constant light for 24 h until end of the trial.

**Table 1:** Composition and nutrient level of the basal diet (air-dry basis).

Ingredients	Content/%	Nutritional level	Content/%
Corn	63.27	Metabolic energy ME (MJ/kg)	12.78
Soybean meal (45% CP)	27.52	Crude protein	19.08
Soybean oil	3.00	Calcium (g/kg)	0.87
Soy protein powder (65% CP)	2.74	Total phosphorus (g/kg)	0.58
Limestone powder	1.47	Non-phytate phosphorus	0.35
Dicalcium phosphate	1.35	Lysine	0.99
L- Lysine·HCl (98%)	0.14	Methionine	0.41
DL-Methionine (98%)	0.08		
Trace mineral Premix^1^	0.01		
Vitamin premix^2^	0.02		
Choline chloride (50%)	0.10		
Sodium chloride	0.30		
Total	100.00		

^1^Trace mineral premix provided the following (per kilogram of diet): 80 mg iron, 40 mg zinc, 8 mg copper, 60 mg manganese, 0.35 mg iodine, and 0.15 mg selenium. ^2^The vitamin premix provided the following (per kilogram of diet): 8,000 IU vitamin A (retinoids), 1,000 IU vitamin D, 20 IU vitamin E, 0.5 mg vitamin K, 2.0 mg vitamin B1, 8.0 mg vitamin B2, 35 mg vitamin B3 (niacin), 3.5 mg vitamin B6 (pyridoxine), 0.01 mg vitamin B12, 10 mg vitamin B5 (pantothenic acid), 0.55 mg vitamin B9 (folic acid), and 0.18 mg vitamin B7 (biotin).

### 2.4 Growth performance and body size trait

At 23 and 42 days of age (day 1 and 19 of the experiment), live- weight and feed weight were measured per replicate to calculate average daily gain (ADG), average daily feed intake (ADFI), average daily feed intake/average daily gain (F/G). On day 42, after 12 h of fasting, one bird was randomly selected from each replicated to measure the body size trait. Body slanting length (BSL) was gauged from the shoulder to the ipsilateral ischial tuberosity. Keel length (KL) was the distance, between the front and rear ends of the keel. Shank girth (SG) was the circumference of the middle of the shin. Shank length (SL) was determined as the straight-line distance from the supra metatarsal joint to the middle of the third and fourth toes. Chest width (CW) was calculated as the distance between two shoulder joints. Chest depth (CD) was measured from the first thoracic vertebra to the front of the keel. Hip bone width (HBW) was the length between the two side waist corners. Waist edge width (WEW) was the maximum width of the two hip joints on the left and right sides. Here, caliper was used to measure shank length, chest width, chest depth, and hip bone width and a measuring tape was used to record body slanting length, keel length, shank girth and waist edge width. All operations were carried out following agricultural standard NY/T823-2020 (16).

### 2.5 Blood collection and slaughter performance measurement

Thirty broilers (one per replicate), the venous blood was drawn from the wing veins of birds, and placed in centrifuge tubes for overnight at 4 °C. Serum was collected after centrifugation (3,000 g, 10 min, at 4 °C), placed into sterile tubes, and kept at −80 °C until analysis. Then, birds were slaughtered. After bloodletting, feather extraction, beak shell and foot skin removed, the weight of carcass, evisceration, semi-evisceration, abdominal fat, breast muscle, leg muscle, wing (a pair), legs (two) were recorded. Next, the slaughter percentage, eviscerated weight percentage, semi-eviscerated weight percentage, abdominal fat percentage, breast muscle percentage, leg muscle percentage, wing weight percentage and leg weight percentage were calculated. The determination method is in accordance with the agricultural industry standard NY/T 823-2004 “Terms and Measurement and Statistical Methods for Poultry Production Performance”.

### 2.6 Relative weight of internal organs calculation

Thirty broilers (one per replicate), the heart, liver, spleen, lung, kidney, thymus, pancreas, intestine, glandular stomach, muscular stomach and bursa of Fabricius were immediately dissected, blotted and weighed. Relative weight of internal organs = weight of organ/live weight × 100%.

### 2.7 Cytokines, immunoglobulins, and antioxidant indicators measurement

Interleukin 4 (IL-4), Interleukin 6 (IL-6), and Interleukin 1β (IL-1β) levels were measured in serum using the enzyme-linked immunosorbent assay (ELISA) kits specific for poultry according to the manufacturer’s instructions (Jiancheng Biotechnology Co. Ltd., Nanjing, China). At the same time, the immunoglobulin A (IgA), immunoglobulin G (IgG), and immunoglobulin M (IgM) levels in serum were determined with ELISA kits specific for chicken according to the manufacturer’s instructions (Shanghai Mlbio Co., Ltd. Shanghai China).

The oxidation-stress related indicators were determined as described by Guo et al. (17). The level of total antioxidant capacity (T-AOC) in bird serum was detected with ELISA kits specific for poultry (Jiancheng Biotechnology Co. Ltd., Nanjing, China). The catalase (CAT) level was measured using the molybdate colorimetric method. The glutathione peroxidase (GSH-Px) level was detected using the 5,5′-dithio-nitrobenzoic acid (DTNB) chromogenic method. Superoxide dismutase (SOD) in serum was measured by the modified pyrogallol autoxidation method. Malondialdehyde (MDA) was measured by the modified thiobarbituric acid reactive substances assay.

### 2.8 Cecal microbiota analysis

The cecal contents were collected and sent to Beijing Biomarker Technologies Co. Ltd. (Beijing, China) for 16S rRNA gene sequencing. Three samples each for five groups.

Total bacterial DNA was extracted from ceca contents (100 mg) using the E.Z.N. ATM Mag-Bind Soil DNA Kit (Omega Bio-Tek, USA), and the extracted DNA was detected by 1% gel electrophoresis. Qubit R 4.0 (ThermoFisher Scientific, USA) was used to determine the DNA concentration. The synthesized primers (338F: ACTCCTACGGGAGGCAGCA; 806R: GGACTACHVGGGTWTCTAAT) were used for PCR amplification of the 16S r RNA gene V3/V4 regions. All of the PCR reactions were conducted in triplicates. The amplified products were quality evaluated by using 2% agarose gel electrophoresis and Qubit^®^ 4.0 fluorescence quantifier. Sequencing libraries were using the TruSeq^®^ Nano DNA Kit (Illumina, USA). The qualified libraries were used for paired-end sequenced (2 × 250 bp) on the Illumina NovaSeq 6,000 platform.

### 2.9 Bioinformatics and statistical analysis

Trimmomatic v0.33 and cutadapt 1.9.1 software was used to remove the adapters, primers, and low-quality sequences from the raw reads, and denoise, double-ended sequence splicing, and removal of chimeric sequences was by the DADA2 plug-in. The processed reads were clustered into Operational Taxonomic Unit (OTUs) with 99% sequence identity. Then, phylogenetic analysis and multiple sequences were performed based on the obtained OTUs. Before conducting the diversity analysis, the rarefaction curves were generated to reflect sequencing depth before diversity analysis. q2-diversity plug-in in QIIME2 2020.6 software was used to analysis microbiota diversity and composition. The significance of differences between groups were analyzed using the Wilcoxon rank-sum test, and the *p-*values were corrected by FDR. The Linear discriminant analysis (LDA) effect size (LEfSe) was conducted by Python Lefse package to search for biomarkers with statistical differences between groups based on LDA > 4 and *p* < 0.05. Statistical analysis was performed using One-way ANOVA in SPSS26.0 (SPSS Inc., USA), and multiple comparisons was conducted by Duncan’s method. Using the ‘corrr’ and ‘ggplot2’ packages in R (R version 4.5.2), the ‘pearson’ method was employed to calculate the microbial correlation matrix and generate a correlation heatmap.

## 3 Results

### 3.1 Growth performance

Table 2 shows the effects of dietary CYRG on broilers’ growth performance in this study. In the trail period, the highest body weight gain was determined in the 0.5% CYRG group (1669.20 g), while body weights between 1429.40 and 1598.60 g were obtained in the 2% CYRG, 1% CYRG, 1.5% CYRG and CON groups, respectively. No difference of ADG, ADFI and F/G between the treatment and the CON groups were observed during the trail periods (*P* > 0.05). However, the 0.5% CYRG and 1.5% CYRG treatment groups had higher ADG, and 0.5% CYRG group had the highest ADG in all trail periods.

**Table 2:** Effects of dietary CYRG on the growth performance in broilers.

Item	CON	CYRG levels				SEM	*p*-value		
		0.5% CYRG	1% CYRG	1.5% CYRG	2% CYRG		ANOVA	Linear	Quadratic
Initial body weight/g	1040.00	1034.80	1035.00	1029.40	1030.60	23.293	1.000	0.899	0.997
Final body weight/g	2596.00	2704.00	2508.00	2628.00	2460.00	50.576	0.603	0.966	0.959
Body weight gain/g	1556.00	1669.20	1473.00	1598.60	1429.40	42.206	0.409	0.981	0.948
ADG/g	81.89	87.85	77.53	84.14	75.23	2.221	0.409	0.981	0.948
ADFI/g	168.74	181.26	166.63	178.37	165.95	2.453	0.453	0.748	0.714
F/G	2.06	2.06	2.15	2.12	2.21	0.038	0.726	0.225	0.829

CYRG, *Chinese yam*—*Rehmannia glutinosa* ‘medicine pair’; CON, control group; 0.5% CYRG, fed with the basal diet supplemented with 0.5% CYRG; 1% CYRG, fed with the basal diet supplemented with 1% CYRG; 1.5% CYRG, fed with the basal diet supplemented with 1.5% CYRG; 2% CYRG, fed with the basal diet supplemented with 2% CYRG; ADG, average daily gain; ADFI, average daily feed intake; F/G, average daily feed intake/average daily gain. *n* = 6.

### 3.2 Body size measurements

The effects of dietary CYRG on body size measurements are presented in Table 3. No difference of the measured body size data was observed among the treatment groups in this study (*P* > 0.05). Several body size measurements, including BSL, KL, AND CD of broilers in the CON group were higher than those in the supplementation CYRG groups, while SG and SL in the dietary CYRG treatment groups were higher than those in the CON group.

**Table 3:** Effects of dietary CYRG on the body size measurements in broilers.

Item/cm	CON	CYRG levels				SEM	*P*-value		
		0.5% CYRG	1% CYRG	1.5% CYRG	2% CYRG		ANOVA	Linear	Quadratic
BSL	20.13	19.79	19.82	19.83	18.77	0.232	0.424	0.691	0.736
KL	15.85	15.67	15.55	15.62	15.48	0.153	0.964	0.645	0.741
SG	5.17	5.58	5.47	5.26	5.32	0.072	0.404	0.765	0.067
SL	10.17	10.58	10.75	10.18	10.34	0.143	0.679	0.923	0.158
CW	9.29	8.96	9.37	9.25	9.10	0.102	0.754	0.943	0.666
CD	8.42	7.78	7.98	8.07	8.36	0.126	0.497	0.435	0.217
HBW	10.68	10.70	10.23	10.18	10.24	0.104	0.290	0.089	0.879
WEW	10.30	9.91	10.87	10.20	9.51	0.191	0.244	0.909	0.734

CYRG, *Chinese yam*—*Rehmannia glutinosa* ‘medicine pair’; CON, control group; 0.5% CYRG, fed with the basal diet supplemented with 0.5% CYRG; 1% CYRG, fed with the basal diet supplemented with 1% CYRG; 1.5% CYRG, fed with the basal diet supplemented with 1.5% CYRG; 2% CYRG, fed with the basal diet supplemented with 2% CYRG; BSL, Body slanting length; KL, Keel length; SG, Shank girth; SL, Shank length; CW, Chest width; CD, Chest depth; HBW, Hip bone width; WEW, Waist edge width. *n* = 6.

### 3.3 Slaughter performance

Table 4 shows the effects of dietary CYRG on the slaughter performance in broilers. Slaughter percentage and semi-evisceration weight percentage of broilers in the dietary addition of CYRG groups were significantly higher than those in the CON group (*P* < 0.05). Meanwhile, the CYRG treatment groups had a higher evisceration weight percentage and leg weight percentage compared with the CON group. Furthermore, abdominal fat percentage, breast muscle percentage, leg muscle percentage, and wing weight percentage were not different among all groups (*P* > 0.05).

**Table 4:** Effects of dietary CYRG on the slaughter performance in broilers.

Item (%)	CON	CYRG levels				SEM	*P*-value		
		0.5% CYRG	1% CYRG	1.5% CYRG	2% CYRG		ANOVA	Linear	Quadratic
Slaughter percentage	89.36^b^	91.88^a^	91.48^a^	90.26^ab^	91.48^a^	0.297	0.024	0.152	0.116
Semi-evisceration weight percentage	82.38^b^	85.71^a^	85.53^a^	84.67^a^	85.97^a^	0.402	0.016	0.016	0.100
Evisceration weight percentage	79.65	80.97	81.06	81.19	80.64	0.281	0.444	0.284	0.132
Abdominal fat percentage	1.93	2.02	2.04	1.91	2.24	0.068	0.600	0.318	0.573
Breast muscle percentage	28.294	27.332	27.381	27.324	28.795	0.445	0.790	0.767	0.238
Leg muscle percentage	18.08	17.47	19.66	17.69	17.51	0.348	0.239	0.699	0.252
Wing weight percentage	9.17	8.38	9.27	8.82	8.54	0.148	0.256	0.428	0.793
Leg weight percentage	28.75	28.96	31.14	29.38	29.29	0.408	0.387	0.608	0.196

CYRG, *Chinese yam*—*Rehmannia glutinosa* ‘medicine pair’; CON, control group; 0.5% CYRG, fed with the basal diet supplemented with 0.5% CYRG; 1% CYRG, fed with the basal diet supplemented with 1% CYRG; 1.5% CYRG, fed with the basal diet supplemented with 1.5% CYRG; 2% CYRG, fed with the basal diet supplemented with 2% CYRG; Slaughter percentage, % = slaughter weight/live weight × 100; Semi-evisceration weight percentage, % = semi-eviscerated weight/live weight × 100; Evisceration weight percentage, % = eviscerated weight/live weight × 100; Abdominal fat percentage, % = abdominal fat weight/(abdominal fat weight + eviscerated weight) × 100; Breast muscle percentage, % = breast muscle weight/eviscerated weight × 100; Leg muscle percentage, % = two legs muscle weight/eviscerated weight × 100; Wing weight percentage, % = a pair of wings weight/eviscerated weight × 100; Leg weight percentage, % = two legs weight/eviscerated weight × 100; ^a,b^ different letters in the same indicator represent significant differences, *n* = 6.

### 3.4 Relative weight of internal organs

As shown in Table 5, no significant difference among the five groups was found for all of the detected of the relative internal organs weight in this study (*P* > 0.05). The relative weight of lung and kidney of the broilers in the CYRG treatment groups were higher than those in the CON group, while glandular stomach and muscular stomach in the in the CYRG treatment groups were lower compared to the CON group.

**Table 5:** Effects of dietary CYRG on the relative weight of internal organs in broilers.

Item (g/kg BW)	CON	CYRG levels				SEM	*P*-value		
		0.5% CYRG	1% CYRG	1.5% CYRG	2% CYRG		ANOVA	Linear	Quadratic
Heart	5.01	5.08	5.43	5.41	4.82	0.120	0.450	0.242	0.860
Liver	18.37	18.06	19.33	17.48	16.71	0.353	0.184	0.558	0.314
Spleen	0.97	0.86	0.99	0.87	0.87	0.040	0.760	0.588	0.994
Lung	4.47	4.51	4.78	5.32	4.95	0.163	0.488	0.106	0.504
Kidney	4.47	5.80	5.17	5.78	5.41	0.193	0.167	0.056	0.390
Thymus	3.65	3.87	3.12	4.05	3.34	0.241	0.773	0.762	0.540
Bursa fabricius	1.93	1.75	2.12	2.37	2.01	0.115	0.550	0.185	0.418
Pancreas	1.72	1.88	1.62	1.70	1.44	0.084	0.587	0.236	0.385
Intestine	27.44	27.16	28.90	28.00	26.97	0.502	0.781	0.596	0.796
Glandular stomach	4.44	3.16	3.87	3.39	3.61	0.246	0.556	0.272	0.486
Muscular stomach	10.22	7.07	8.43	8.80	9.89	0.455	0.191	0.421	0.083

CYRG, *Chinese yam*—*Rehmannia glutinosa* ‘medicine pair’; CON, control group; 0.5% CYRG, fed with the basal diet supplemented with 0.5% CYRG; 1% CYRG, fed with the basal diet supplemented with 1% CYRG; 1.5% CYRG, fed with the basal diet supplemented with 1.5% CYRG; 2% CYRG, fed with the basal diet supplemented with 2% CYRG. *n* = 6.

### 3.5 Serum cytokines

As shown in Table 6, the serum concentrations of IL-4 in the 0.5, 1, and 1.5% CYRG treatment groups were significantly higher than those in the CON, and 2% groups (*p* < 0.05). The serum concentration of IL-6 in the CON group were significantly higher than those in the 0.5, 1, and 2% CYRG groups (*p* < 0.05). In addition, the serum concentration of IL-1β in the 2% CYRG group were significantly higher than those in the other four groups (*p* < 0.05).

**Table 6:** Effects of dietary CYRG on the serum cytokine levels in broilers.

Item (ng/L)	CON	CYRG levels				SEM	*P*-value		
		0.5% CYRG	1% CYRG	1.5% CYRG	2% CYRG		ANOVA	Linear	Quadratic
IL-4	27.41^b^	51.68^a^	46.27^a^	46.26^a^	23.96^b^	2.605	<0.001	<0.001	0.012
IL-6	51.68^a^	11.68^d^	28.96^c^	45.60^ab^	32.40^bc^	3.138	<0.001	0.579	<0.001
IL-1β	55.52^bc^	40.46^c^	45.58^bc^	57.70^b^	75.43^a^	2.967	<0.001	0.005	0.000

CYRG, *Chinese yam*—*Rehmannia glutinosa* ‘medicine pair’; CON, control group; 0.5% CYRG, fed with the basal diet supplemented with 0.5% CYRG; 1% CYRG, fed with the basal diet supplemented with 1% CYRG; 1.5% CYRG, fed with the basal diet supplemented with 1.5% CYRG; 2% CYRG, fed with the basal diet supplemented with 2% CYRG; ^a,b^ different letters in the same indicator represent significant differences, *n* = 6.

### 3.6 Serum immunoglobulins

The effects of dietary CYRG on the concentrations of serum immunoglobulins in broilers are shown in Table 7. The serum concentration of IgG in the 1.5% CYRG group was significantly higher than that in the CON group (*p* < 0.05). In addition, the serum IgM concentrations in the 1.5 and 2% CYRG groups were higher than that in the CON group (*p* < 0.05). Meanwhile, no differences in serum IgA concentrations were found among all the groups (*p* > 0.05).

**Table 7:** Effects of dietary CYRG on the serum immunoglobulin levels in broilers.

Item (mg/mL)	CON	CYRG levels				SEM	*P*-value		
		0.5% CYRG	1% CYRG	1.5% CYRG	2% CYRG		ANOVA	Linear	Quadratic
IgA	2.28	3.01	2.65	3.55	2.80	0.171	0.192	0.033	0.811
IgG	7.08^b^	8.74^ab^	7.83^ab^	9.67^a^	9.34^ab^	0.313	0.035	0.011	0.882
IgM	3.08^b^	4.66^ab^	5.27^ab^	6.22^a^	6.25^a^	0.374	0.026	0.064	0.087

CYRG, *Chinese yam*—*Rehmannia glutinosa* ‘medicine pair’; CON, control group; 0.5% CYRG, fed with the basal diet supplemented with 0.5% CYRG; 1% CYRG, fed with the basal diet supplemented with 1% CYRG; 1.5% CYRG, fed with the basal diet supplemented with 1.5% CYRG; 2% CYRG, fed with the basal diet supplemented with 2% CYRG; ^a,b^ different letters in the same indicator represent significant differences, *n* = 6.

### 3.7 Serum antioxidant enzyme activities

As shown in Table 8, the activities of CAT, GSH-Px, and T-AOC in the serum of all experimental groups were higher than those of the CON group, while the activity of MDA was on the contrary. Meanwhile, the activity of CAT in the 0.5% CYRG group was significantly higher than that in the CON group (*p* < 0.05). The activities of GSH-Px and T-AOC in the 0.5% CYRG and 2% CYRG groups were significantly higher than those in the CON group (*p* < 0.05). The level of MDA in the 0.5% CYRG group was significantly lower than that in the CON group (*p* < 0.05). Among all five groups, no differences in serum SOD were determined (*P* > 0.05).

**Table 8:** Effects of dietary CYRG on the serum antioxidant enzyme activities in broilers.

Item (U/mL)	CON	CYRG levels				SEM	*P*-value		
		0.5% CYRG	1% CYRG	1.5% CYRG	2% CYRG		ANOVA	Linear	Quadratic
SOD	255.43	269.14	265.71	248.79	270.00	3.130	0.120	0.440	0.028
CAT	7.32^b^	12.36^a^	9.61^ab^	10.68^ab^	11.44^ab^	0.534	0.016	0.157	0.754
GSH-Px	561.33^b^	657.25^a^	623.73^ab^	636.06^ab^	661.05^a^	11.561	0.029	0.043	0.070
MDA	9.67^a^	4.71^b^	6.54^ab^	5.80^ab^	8.98^ab^	0.590	0.020	0.036	0.122
T-AOC	4.07^c^	15.91^a^	8.14^bc^	7.77^bc^	12.95^ab^	1.100	0.001	0.013	0.340

CYRG, *Chinese yam*—*Rehmannia glutinosa* ‘medicine pair’; CON, control group; 0.5% CYRG, fed with the basal diet supplemented with 0.5% CYRG; 1% CYRG, fed with the basal diet supplemented with 1% CYRG; 1.5% CYRG, fed with the basal diet supplemented with 1.5% CYRG; 2% CYRG, fed with the basal diet supplemented with 2% CYRG; SOD, superoxide dismutase; CAT, catalase; GSH-Px, glutathione peroxidase; MDA, malondialdehyde; T-AOC, total antioxidant capacity; ^a,b^ different letters in the same indicator represent significant differences, *n* = 6.

### 3.8 Correlation between serum cytokines or immunoglobulins and antioxidant indexes

**Figure 1:**
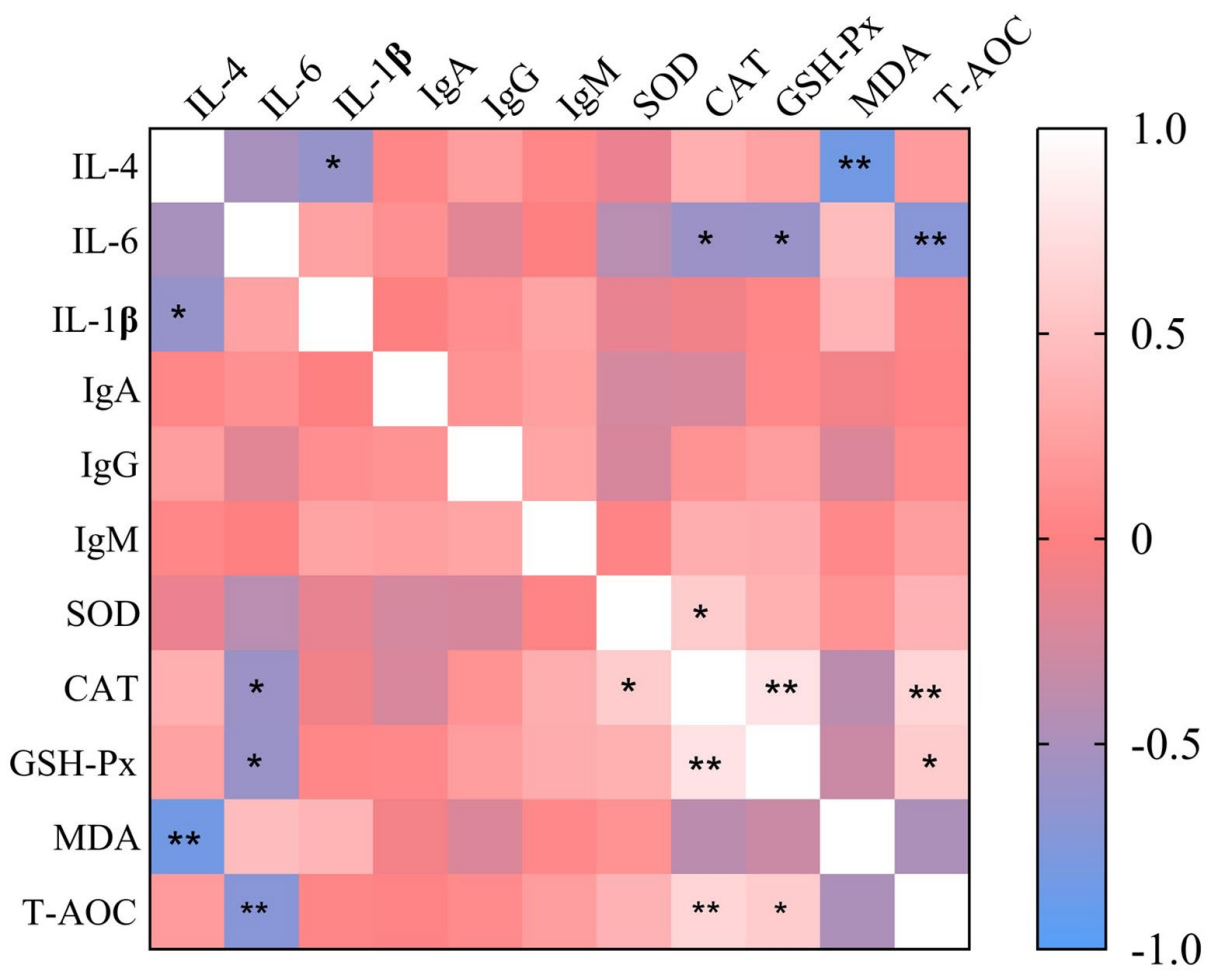
Correlation of serum cytokines, immunoglobulins, and antioxidant indexes. IL-4, Interleukin 4; IL-6, Interleukin 6; IL-1B, Interleukin 1ß; IgA, immunoglobulin A; IgG, immunoglobulin G; IgM, immunoglobulin M; SOD, superoxide dismutase; CAT, catalase; GSH-Px, glutathione peroxidase; MDA, malondialdehyde; T-AOC, total antioxidant capacity. *Represent *p* < 0.05, ** Represent *p* < 0.01.

The analysis revealed that IL-4 level in the serum of broiler was negatively associated with IL-1β and MDA levels (*p* < 0.05) (Figure 1). Moreover, serum IL-6 level was negatively correlated with antioxidant indexes, such as CAT, GSH-Px, and T-AOC levels (*p* < 0.05). And CAT level has been positively associated with SOD, GSH-Px, and T-AOC levels (*p* < 0.05). GSH-Px level was positively correlated with T-AOC level (*p* < 0.05). CYRG intervention significantly elevated the levels of CAT, GSH-Px, and T-AOC, combined with the MDA level declined. Meanwhile, CYRG intervention significantly elevated the level of anti-inflammatory factor IL-4 and immunoglobulin (IgA, IgG, and IgM), and declined the inflammatory factors IL-6, and IL-1β. Therefore, CYRG inhibit the release of inflammatory factors, and elevate the immune and antioxidant activities.

### 3.9 Composition and diversity of caca microbial flora

**Figure 2:**
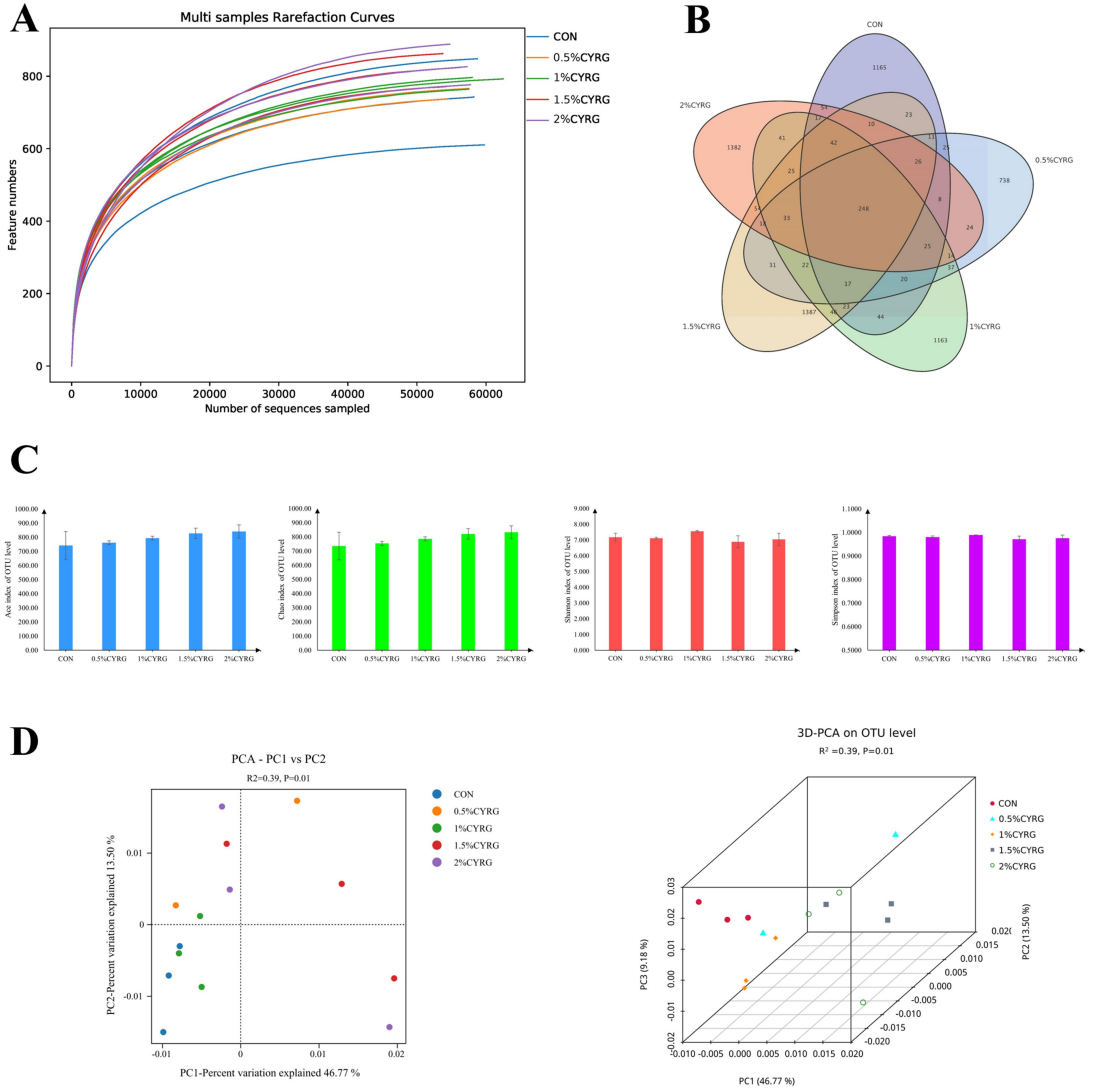
Effects of *Chinese yam*—*Rehmannia glutinosa* ‘medicine pair’s (CYRG) on cecal microbiota diversity in broilers. **(A)** Microbial rarefaction curves based on OTU level were used to assess the depth of coverage for each sample. Each treatment samples were distinguished by different colors of lines. **(B)** Venn diagram of OTUs level. **(C)** The alpha diversity parameters including Ace, Chao, Shannon, and Simpson index. **(D)** Principal component analysis (PCA) scores plot of the samples.

Clustering of microbes in the ceca microbiota was observed among the five groups (Figure 2). An average of 58,078 clean reads obtained from each ceca sample. Figure 2A showed that the sequence number was more than 50,000 reads, while the OTU number kept unchanged, indicated that the sequencing depth was sufficient to adequately reflect the microbial community composition of the ceca samples. Among the 6,773 clustered OTUs, 248 OTUs were shared by all the five treatment groups (Figure 2B), and the CON, 0.5% CYRG, 1% CYRG, 1.5% CYRG, and 2% CYRG groups were found to contain 1758, 1,297, 1817, 2016, and 2021 distinct OTUs, respectively. And as shown in Figure 2B, 1,165, 738, 1,163, 1,387, and 1,382 unique OTUs were identified in the CON, 0.5% CYRG, 1% CYRG, 1.5% CYRG, and 2% CYRG groups, respectively. The alpha diversity parameters, including Ace, Chao, Shannon, and Simpson index (Figure 2C and Table 9), were not different among the five treatment groups in this paper (*p* > 0.05). *R* value calculated by ANOSIM was above 0, indicating there were greater difference between groups. The principal components analysis (PCA) (Figure 2D) showed that there was greater difference between groups (*p* = 0.01), and CYRG supplementation had distinguishable clustering with the CON group while the principal component axes PC1, PC2, and PC3 explained 46.77, 13.50, and 9.18% of the total variation, respectively.

**Table 9:** Effects of dietary CYRG on cecal microbiota alpha diversity paraments in broilers (OTU level).

Group	Richness index		Diversity index		Coverage/%
	ACE	Chao	Shannon	Simpson	
CON	742.48 ± 98.62	735.66 ± 97.52	CON	0.9841 ± 0.0032	0.9997 ± 0.0000816
0.5% CYRG	761.71 ± 13.67	753.98 ± 14.29	0.5% CYRG	0.9808 ± 0.0047	0.9997 ± 0.0001414
1% CYRG	794.37 ± 13.52	786.6 ± 14.11	1% CYRG	0.9896 ± 0.0005	0.9996 ± 0.0000471
1.5% CYRG	827.64 ± 36.77	821.23 ± 37.10	1.5% CYRG	0.9717 ± 0.0126	0.9996 ± 0.0000471
2% CYRG	841.25 ± 46.46	832.7 ± 45.79	2% CYRG	0.9762 ± 0.0127	0.9996 ± 0.0000943

CYRG, *Chinese yam*—*Rehmannia glutinosa* ‘medicine pair’; CON, control group; 0.5% CYRG, fed with the basal diet supplemented with 0.5% CYRG; 1% CYRG, fed with the basal diet supplemented with 1% CYRG; 1.5% CYRG, fed with the basal diet supplemented with 1.5% CYRG; 2% CYRG, fed with the basal diet supplemented with 2% CYRG. *n* = 3.

**Figure 3:**
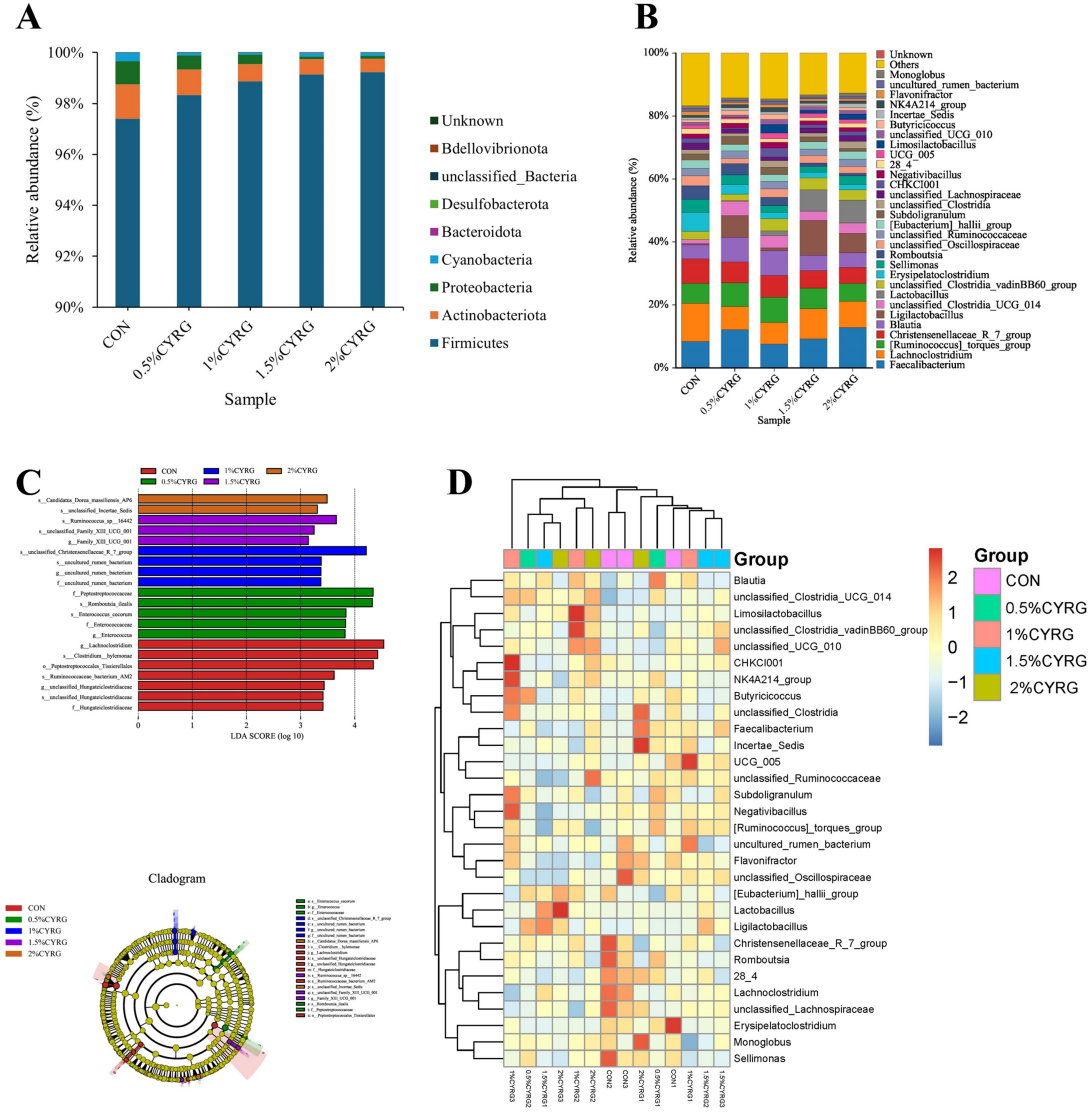
Effects of *Chinese yam*—*Rehmannia glutinosa* ‘medicine pair’s (CYRG) on cecal microbiota composition in broilers. **(A)** Microbial composition at the phylum level. **(B)** Microbial composition at the genus level. **(C)** Difference between the cecal microbiota of the five groups of broilers were determined by linear discriminant analysis effect size (LEfSe). **(D)** Clustering heatmap of the cecal microbial in each sample at the genus level. Red represents positive correlation and blue indicates negative correlation.

Taxonomic unit analysis revealed that the dominant phyla (Figure 3A and Table 10) of the five groups included Firmicutes (97.39, 98.33, 98.87, 99.14, 99.23%), which is in absolute dominance, followed by Actinobacteriota (1.34, 1.01, 0.67, 0.60, 0.53%), and Proteobacteria (0.93, 0.55, 0.37, 0.11, 0.12%). The relative abundance of Actinobacteriota in 1.5% CYRG and 2% CYRG groups were significantly declined compared with that in the CON, 0.5% CYRG, and 1% CYRG groups (*P* < 0.05) (Table 10). And CYRG supplementation significantly declined the relative abundance of Cyanobacteria compared to the CON group. The relative abundance of Firmicutes, Proteobacteria, and Bacteroidota were not affected by CYRG supplementation (*p* > 0.05) (Table 10). At the genus level (Figure 3B and Table 11), the dominant microorganisms in the five groups were *Faecalibacterium* (8.34, 12.16, 7.59, 9.20, 12.69%), *Lachnoclostridium* (12.16, 7.38, 6.87, 9.59, 8.35%), *[Ruminococcus]_torques_group* (6.36, 7.47, 8.10, 6.52, 5.72%), *Christensenellaceae_R_7_group* (7.70, 6.67, 6.91, 5.59, 5.04%), and *Blautia* (4.53, 7.90, 7.89, 4.94, 4.62%). Compared with the CON group, 0.5% CYRG and 1% CYRG supplementation significantly declined the relative abundance of *Lachnoclostridium*, and increased the abundance of *unclassified_Clostridia_UCG_014* (*p* < 0.05) (Table 11). The birds of 1.5% CYRG group had more *Ligilactobacillus* compared to the CON and 1% groups (*p* < 0.05). Contrast to the CON, 1% CYRG and 1.5% CYRG birds had fewer *Sellimonas*, and 1.5% CYRG and 2% CYRG birds had fewer *Romboutsia* (*p* < 0.05). In addition to that, we can find that compared to the CON and 0.5% CYRG groups, 1% CYRG supplementation decreased the number of *unclassified_Lachnospiraceae* in the caecum of broilers, while increased significantly the number of *Limosilactobacillus* (*p* < 0.05) (Table 11).

**Table 10:** Effects of dietary CYRG on cecal microbiota composition in broilers (the top five, at phylum level).

Microorganism	CON	CYRG levels				SEM	*p*-value
		0.5% CYRG	1% CYRG	1.5% CYRG	2% CYRG		
Firmicutes	97.39	98.33	98.87	99.14	99.23	0.290	0.213
Actinobacteriota	1.34^a^	1.01^ab^	0.67^ab^	0.60^b^	0.53^b^	0.113	0.075
Cyanobacteria	0.33^a^	0.11^b^	0.08^b^	0.14^b^	0.11^b^	0.032	0.045
Proteobacteria	0.93	0.55	0.37	0.11	0.12	0.175	0.591
Bacteroidota	0.00	0.01	0.00	0.01	0.00	0.002	0.293

CYRG, *Chinese yam*—*Rehmannia glutinosa* ‘medicine pair’; CON, control group; 0.5% CYRG, fed with the basal diet supplemented with 0.5% CYRG; 1% CYRG, fed with the basal diet supplemented with 1% CYRG; 1.5% CYRG, fed with the basal diet supplemented with 1.5% CYRG; 2% CYRG, fed with the basal diet supplemented with 2% CYRG; ^a,b^ different letters in the same indicator represent significant differences, *n* = 3.

**Table 11:** Effects of dietary CYRG on cecal microbiota composition in broilers (at genus level).

Microorganism	CON	CYRG levels				SEM	*p*-value
		0.5% CYRG	1% CYRG	1.5% CYRG	2% CYRG		
*Faecalibacterium*	8.34	12.16	7.59	9.20	12.69	1.246	0.681
*Lachnoclostridium*	12.16^a^	7.38^b^	6.87^b^	9.59^ab^	8.35^ab^	0.619	0.011
[*Ruminococcus*]_torques_group	6.36	7.47	8.10	6.52	5.72	0.433	0.471
Christensenellaceae_R_7_group	7.70	6.67	6.91	5.59	5.04	0.402	0.194
*Blautia*	4.53	7.90	7.89	4.94	4.62	0.626	0.187
*Ligilactobacillus*	0.36^b^	6.76^ab^	0.96^b^	10.96^a^	6.28^ab^	1.467	0.046
Unclassified_*Clostridia*_UCG_014	1.31^b^	4.58^a^	3.86^a^	2.94^ab^	3.24^ab^	0.388	0.033
*Lactobacillus*	0.03	0.09	1.41	6.81	7.48	1.613	0.426
Unclassified_*Clostridia*_vadinBB60_group	2.55	2.06	3.96	3.80	3.24	0.381	0.577
*Erysipelatoclostridium*	6.06^a^	3.09^b^	2.01^b^	1.80^b^	1.76^b^	0.558	0.017
*Sellimonas*	4.18^a^	3.07^ab^	2.14^b^	1.90^b^	2.69^ab^	0.297	0.047
*Romboutsia*	4.26^a^	3.63^ab^	2.60^ab^	1.05^b^	0.80^b^	0.501	0.041
Unclassified_Oscillospiraceae	3.12	1.69	2.70	2.35	2.17	0.289	0.708
Unclassified_Ruminococcaceae	2.41	2.38	2.43	2.09	2.37	0.143	0.960
[*Eubacterium*]_hallii_group	2.67	2.06	2.17	2.28	2.41	0.202	0.931
*Subdoligranulum*	1.86	2.51	2.22	1.56	0.93	0.236	0.301
Unclassified_*Clostridia*	1.37	1.07	2.10	1.23	2.27	0.265	0.577
Unclassified_Lachnospiraceae	2.11^a^	1.20^ab^	1.14^b^	1.59^ab^	1.74^ab^	0.140	0.045
CHKCI001	1.52	0.43	2.83	1.13	1.38	0.408	0.543
*Negativibacillus*	1.43	1.56	1.78	1.20	1.24	0.137	0.712
28_4	1.66	1.38	1.09	1.04	1.36	0.116	0.447
UCG_005	1.10	0.47	1.87	1.31	1.15	0.301	0.807
*Limosilactobacillus*	0.03^b^	0.08^b^	2.70^a^	0.95^ab^	1.74^ab^	0.384	0.048
Unclassified_UCG_010	0.84	0.66	1.63	1.20	1.14	0.180	0.586
*Butyricicoccus*	0.78	1.46	1.64	0.33	0.83	0.194	0.173
Incertae_Sedis	0.79	0.90	0.80	0.72	1.33	0.122	0.548
NK4A214_group	0.67	0.97	1.36	0.66	0.81	0.126	0.386
Flavonifractor	1.24	0.76	0.96	0.71	0.72	0.103	0.448
Uncultured_rumen_bacterium	0.90	0.65	1.11	0.60	0.78	0.073	0.138
*Monoglobus*	0.90	0.77	0.63	0.72	1.00	0.070	0.483
Others	16.77	14.22	14.56	13.27	12.68	0.565	0.134

CYRG, *Chinese yam*—*Rehmannia glutinosa* ‘medicine pair’; CON, control group; 0.5% CYRG, fed with the basal diet supplemented with 0.5% CYRG; 1% CYRG, fed with the basal diet supplemented with 1% CYRG; 1.5% CYRG, fed with the basal diet supplemented with 1.5% CYRG; 2% CYRG, fed with the basal diet supplemented with 2% CYRG; ^a,b^ different letters in the same indicator represent significant differences, *n* = 3.

The LEfSe results (Figure 3C) showed that *Lachnoclostridium*, *Clostridium hylemonae*, *Peptostreptococcales Tissierellales*, *Ruminococcaceae bacterium AM2*, *unclassified Hungateiclostridiaceae*, and *Hungateiclostridiaceae* were enriched in the CON group. The microbiota in the 0.5% CYRG group were more abundant in *Peptostreptococcaceae*, *Romboutsia ilealis*, *Enterococcus cecorum*, *Enterococcaceae*, and *Enterococcus*. The relative abundance of *unclassified Christensenellaceae R7 group* and *uncultured_rumen_bacterium* was increased in the 1% CYRG group. The 1.5% CYRG group was significantly populated with *Ruminococcus* sp. *16,442*, *unclassified Family XII UCG 001*, and *Family XII UCG 001*. While *Candidatus Dorea massiliensis AP6* and u*nclassified Incertae Sedis* were highly represented in 2% CYRG group.

Figure 3D showed the variation in species composition and their species abundance distribution trends among groups. The CON group was significantly populated with *Lachnoclostridium, Faecalibacterium, Christensenellaceae_R_7_group, [Ruminococcus]_torques_group, Erysipelatoclostridium*, and *Blautia. Lactobacillus, Faecalibacterium, Blautia, Lachnoclostridium*, and *[Ruminococcus]_torques_group* were greatly enriched in the 0.5% CYRG group. While *[Ruminococcus]_torques_group, Blautia, Faecalibacterium, Christensenellaceae_R_7_group*, and *Lachnoclostridium* were highly represented in 1% CYRG group. The 1.5% CYRG group were populated with *Ligilactobacillus, Lachnoclostridium, Faecalibacterium, Lactobacillus,* and *[Ruminococcus]_torques_group*. The relative abundance of *Faecalibacterium, Lachnoclostridium, Lactobacillus, and Ligilactobacillus* was increased in 2% CYRG group.

### 3.10 Correlation between cecal microorganisms, cytokines, immunoglobulin, and antioxidant indexes

**Figure 4:**
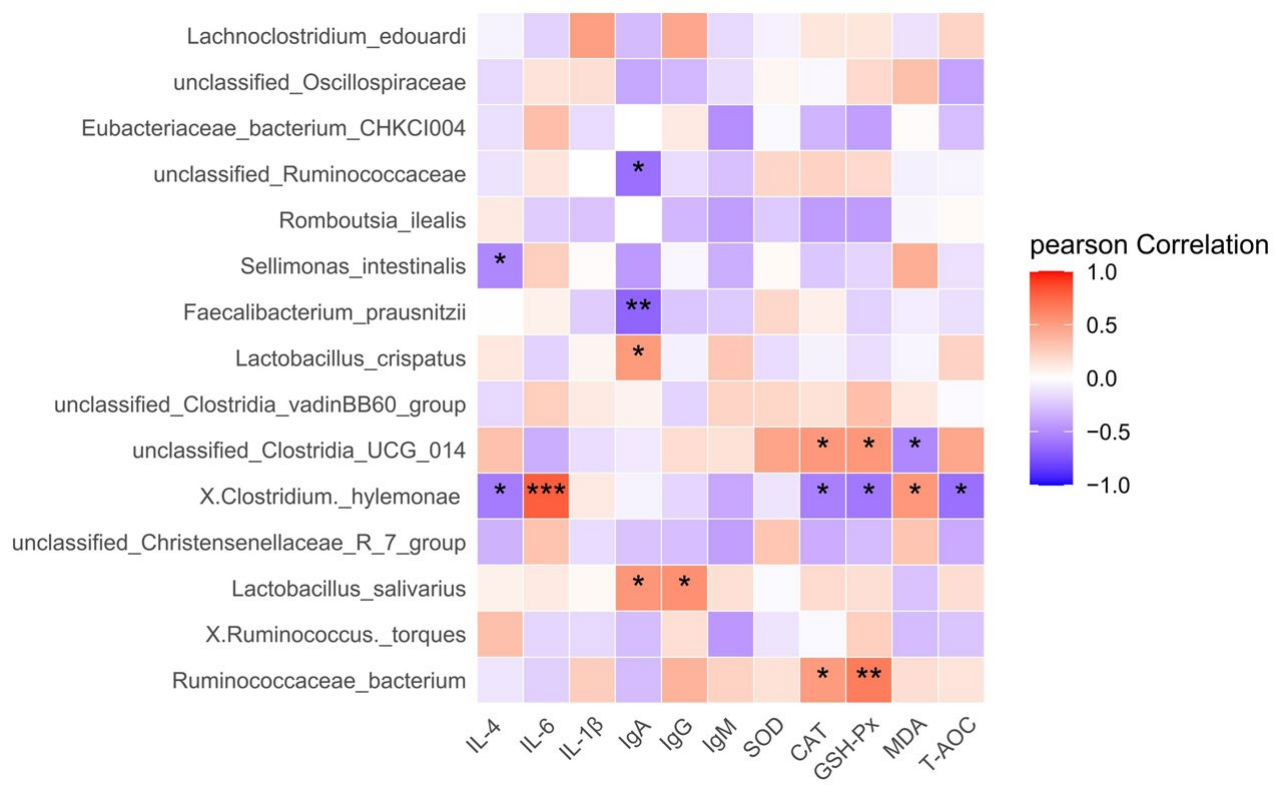
Correlation analysis of intestinal flora. IL-4, Interleukin 4; IL-6, Interleukin 6; IL-1ß, Interleukin 1; IgA, Immunoglobulin A; IgG, Immunoglobulin G; IgM, Immunoglobulin M; SOD, Superoxide Dismutase; CAT, Catalase; GSH-Px, Glutathione Peroxidase; MDA, Malondialdehyde; T-AOC, Total Antioxidant Capacity. *Represent *p* < 0.05, ** Represent *p* < 0.01.

The correlation analysis revealed that *Clostridium hylemonae* in the cecal of broilers was negatively associated with anti-inflammatory cytokines IL-4, and positively correlated with inflammatory factors IL-6 (*P* < 0.01) (Figure 4). *Sellimonas intestinalis* has been negatively associated with IL-4 (*P* < 0.05). IgA was positively associated with *Lactobacillus salivarius* and *Lactobacillus_crispatus* (*P* < 0.05), and negatively correlated with *unclassified Ruminococcaceae* and *Faecalibacterium prausnitzii* (*P* < 0.01). Meanwhile, *Lactobacillus salivarius* was positively associated with IgG (*P* < 0.05). *Unclassified Clostridia ucG 014* and *Ruminococcaceae bacterium* were positively correlated with CAT and GSH-Px, while *Unclassified Clostridia ucG 014* was negatively correlated with MDA. Moreover, *Clostridium hylemonae* was negatively correlated with antioxidant indexes, such as CAT, GSH-Px, and T-AOC. CYRG intervention significantly elevated the relative abundance of *Unclassified Clostridia ucG 014* and Limosilactobacillus, combined with the structural changes of intestinal flora. Therefore, we speculate that CYRG may be by increasing the proportion of beneficial bacteria in the intestine to enhance the anti-inflammatory cytokines and immunoglobulin production, inhibit the release of Pro-inflammatory cytokines, and elevate antioxidant activity. The mechanism of CYRG relieving intestinal inflammation in broiler needs to be further analyzed.

## 4 Discussion

In actual production, farmers usually adopt the mode of high-density broiler breeding to meet rising global chicken demand (7). High density was related to abnormal broiler behavior, dermatitis, poor feathering and soiling, and more importantly, increased oxidative damage which in turn leads to low growth performance and weak immune function (18–21). Chinese herbs exhibited immunomodulatory, anti-inflammatory, and anti-oxidant activities in different experimental models (7, 22–24). Traditional Chinese plants that are both medicinal and edible, *Chinese yam* and *Rehmannia glutinosa* have been used for a long history. The ‘medicine pair’ is a combination of two herbs to enhance the efficacy of the medicine, improve the therapeutic effect, and reduce side effects. The aim of this study was to evaluate the application potential of *Chinese yam* and *Rehmannia glutinosa* ‘medicine pair’ in broiler chicken farming. Therefore, we made our first attempt to add CYRG to the broiler diet. Through a short-term feeding trial, we monitored the effects of CYRG on the growth performance, slaughter performance and other indicators of broilers, providing a basis for the application of CYRG in broiler breeding.

### 4.1 Growth performance, body size measurement, slaughter performance, and relative weight of internal organs

Pan et al. (24) found that supplemental 2% *Chinese yam* significantly increased ADG and decreased F/G in weaned piglets. Basal diet supplemented with *Radix rehmanniae preparate polysaccharide* (RRPP) did not affect the broilers’ growth performance during 1–21 days, while during 22–35 days and 1–35 days periods, 600 mg/kg and 900 mg/kg RRPP linearly improved the body weight gain and feed conversion ratio. *Rehmannia glutinosa* oligosaccharides significantly increased the body weight, pancreatic index, thymus index, and colon length of mice by lipopolysaccharide (LPS)-induced (25). *Radix rehmanniae preparate* significantly decreased the liver, spleen, kidney, pancreas and fat indexes of diabetic mice, and there was no significant difference in heart and lung indexes (26). 0.5 g/kg *Chinese yam* polysaccharides (CYP) improved the thymus index and spleen index of broilers at 28 and 48 days, and improved the thymus index at 48 days (27). *Yam* treatment significantly decreased the liver index of carbon tetrachloride-induced hepatic fibrosis rats (28). Gavaged administration with 800 mg/kg/d polysaccharides fraction, DOP-2, prepared from *Dioscorea opposita thunb* significantly improved the thymus and spleen indexes of immunosuppressed mice (29). Zhang et al. (3) reported that 0.1% *Chinese yam* polysaccharide copper complex increased remarkedly ADG, slaughter percentage, semi-evisceration weight percentage, evisceration weight percentage, breast percentage, leg muscle percentage and decreased FCR in broilers. 500 mg/kg *Chinese yam* polysaccharide improved live weight, half-eviscerated carcass percentage, eviscerated carcass percentage, and thigh muscle percentage of broilers (7). 250 mg/kg and 500 mg/kg *Chinese yam* polysaccharide improved the thigh muscle percentage (9). Genistein extracted from soy plants increased the broilers’ body weight gain, tibial length, tibial width and slaughter performance and decreased the feed conversion ratio (FCR) (30). Supplementation betaine reduced the feed intake, and 2.25 g/kg betaine improved the weight gain, the carcass weight, breast yield, intestinal length and weight and reduced fat weight of Japanese quails. And they also found there were no difference among groups in body length, shank length, shank diameter, and keel bone length or breast width (31). We found dietary addition CYRG improved the broilers’ slaughter percentage and semi-evisceration weight percentage, while the growth performance, body size trait, and relative organ weight were not altered in this study. We speculated that the reasons for the differences in the above research results might be related to factors such as the species of the test subjects, the duration of the test, and the dose of additives used.

Studies indicate that, compared with single drug administration, the combined use of ‘medicine pair’ can promote the growth, immunity and antioxidant capacity of animals. This is because there is a synergistic effect between the medicine pair, and it can also enhance intestinal health and nutrient absorption by promoting the proliferation of beneficial bacteria, thereby inhibiting the growth of pathogenic bacteria.

### 4.2 Serum cytokines, immunoglobulins, and antioxidant indicators

In this study, the addition of CYRG in diet can enhance the immunity of broilers by improving the concentrations of IL-4, IgA, IgG, and IgM in serum and declining the levels of serum IL-6 and IL-1β, and we recommend the supplementation of 0.5% CYRG in broiler diets. Deng et al. (9) found that 0.50 g/kg *Chinese yam* polysaccharides supplementation in diets improved significantly the serum IL-4, IL-6, IgA, IgG, and IgM levels at 28 days. The levels of serum immunoglobulin IgG, IgM and Newcastle disease antibody were significantly reversed by *Chinese yam* peel supplementation with diets in intramuscular injection of cyclophosphamide (CTX) broilers (32). Zhang et al. (33) found that a non-starch polysaccharide (CYP-A) isolated from *Chinese yam* suppressed pro-inflammatory cytokine production in colitis symptoms mice induced by dextran sulfate sodium (DSS) and reduced oxidative stress. *Rehmannia glutinosa* polysaccharide (RGP) reduced the gene and protein expression levels of pro-inflammatory factors such as TNF-*α*, IL-1β, IL-6, and caspase-1, while upregulated the gene expression levels of the anti-inflammatory factor IL-10 in LPS-induced mice (34). Similar to our research findings. *Chinese yam* and *Rehmannia glutinosa* could increase the levels of serum cytokines and immunoglobulin, thereby enhancing the body’s disease resistance and immune function. Numerous studies indicate that natural botanical are potential antioxidants (35, 36) and antioxidants are the substances that protect organisms against damage caused by oxidation. In present study, an evaluation about the effects of the supplementation CYRG in broilers’ diets on serum antioxidative indicators demonstrated that dietary CYRG significantly increased the levels of CAT, GSH-Px, T-AOC, and decreased the levels of MDA of broilers (0.5% CYRG group), which revealed that CYRG had strong antioxidant activity. A previous study shown that dietary supplementation with 500 mg/kg *Chinese yam* polysaccharide can significantly improve the contents of serum T-AOC, T-SOD, glutathione peroxidase (GPX), and glutathione s-transferase (GST) in broilers (*p* < 0.05) (7). Chen et al. (37) found that supplementation with 1.6% *Chinese yam* by-product in juvenile fish diets could significantly improve the levels SOD and GSH (*p* < 0.05), meanwhile, 0.4 and 1.6% *Chinese yam* by-product significantly declined the levels of MDA, thereby, *Chinese yam* by-product could protect liver and intestine health. *Rehmannia glutinosa* oligosaccharides elevated significantly the activities of intestine SOD, GSH-Px, and CAT, and decreased the content of MDA in LPS-induced mice (25). Qiao et al. (34) found LPS challenge induced pronounced oxidative damage in mice, elevating MDA and CAT levels, and *Rehmannia glutinosa* polysaccharide treatment significantly attenuated these changes, reduced MDA accumulation and normalized CAT activity, improved the redox balance. These studies have demonstrated that both *Chinese yam* and *Rehmannia glutinosa* can enhance the antioxidant function of the animal body. In this study, it was found that the combination of *Chinese yam* and *Rehmannia glutinosa* also has the ability to enhance the animal’s antioxidant capacity, moreover, the ‘medicine pair’ can exert a synergistic effect. Our research found adding 0.5% CYRG could improve the antioxidant function of broilers, mainly manifested in that 0.5% CYRG significantly increased the total antioxidant capacity, glutathione peroxidase and hydrogen peroxide enzyme levels in the serum, and significantly reduced the malondialdehyde level.

### 4.3 Composition and diversity of ceca microorganisms

Composition of intestinal microbiota is a vital determinant of intestinal health. The intestinal microbiota was responsible for converting food into nutrients and energy (38), and modulate the overall health and productiveness in poultry (21, 39). The chicken cecum is considered to be the most important part in the distal intestine and is the greatest concentration of intestinal microorganisms in mature chickens (40). Digestion in the cecum is associated with cecal microbes, therefore, the diversity and composition of the intestinal microbiota are key elements to maintain the intestine health (41–43). In this study, the alpha diversity was not affected by dietary CYRG, while the results of beta diversity analysis showed significant differences between groups. Similar to our research results, Chen et al. (44) found that the alpha diversity indexes of caca microbes were not affected by supplementation with 300 and 600 mg/kg Chlorogenic acid in a basal diet in Hy-line brown pullets. Our results of the PCA analysis indicted a distinction in the ceca microbial community composition among the five treatment groups. Firmicutes was the most abundant phylum with the largest proportion in this study, followed by Actinobacteriota and Proteobacteria, which consistent with the other report (38, 45). The result support other study (46) that Firmicutes, Bacteroidetes, Proteobacteria, Actinobacteria, and Cyanobacteria were the major microbial groups. Microbiota analysis revealed that birds fed diets supplementation with 0.5, 1, 1.5 and 2% CYRG increased the abundance of cecal Firmicutes by 0.97, 1.52, 1.80, and 1.89% and decreased the proportion of Actinobacteriota, Proteobacteria, and Cyanobacteria by 24.63, 50, 55.22, and 60.45%, 40.86, 60.22%, 88.17, and 87.10, 66.67, 75.76, 57.58, and 66.67%, respectively, compared with the CON group. El Kaoutari et al. (47) reported that many members of Firmicutes could encoded carbohydrate-active enzymes to the hydrolysis and the utilization of carbohydrates. And, members of Firmicutes are involved in the degradation of insoluble fibers in food digestion (48). Meanwhile, Turnbaugh et al. (49) found Firmicutes can generate more harvestable energy. In this study, CYRG supplementation increased the abundance of Firmicutes, which indicate that CYRG may enhance the energy intake capacity of broiler chickens. Reid et al. (50) discovered that Proteobacteria members are associated with cellulose activity. Proteobacteria is the largest phylum of bacteria and members of Proteobacteria are all Gram-negative bacteria, including many opportunistic pathogens (51), such as *Escherichia coli*, *Escherichia*, *Salmonella*, *Vibrio*, *Helicobacter*, *Shigella*, *Pseudomonas aeruginosa*, *Vibrio cholerae*, *Yersinia pestis*, *Neisseria meningitidis*, *Neisseria gonorrhoeae*, *Campylobacter jejuni*, *Helicobacter pylori*, etc. and its abundance can significantly increase in the case of a disease. In the present study, the CYRG diet reduced the relative abundance of Proteobacteria, indicating that CYRG can improve intestinal health by reducing Proteobacteria in the cecum. Wu et al. (52) reported that certain bloom-forming cyanobacteria produced microcystins, which can induce toxicity in various organs, such as renal toxicity, reproductive toxicity, cardiotoxicity, and immunosuppressive effects. And they also found the toxicity of microcystins on the gastrointestinal tract is multidimensional, it not only can affect gastrointestinal barrier function but also shift the gut microbiota structure in different gut regions, and it can inhibit the release of inflammatory cytokines, then affects the expression of immune-related genes in the intestine (52). In this study, we found that adding CYRG to the diet reduced the relative abundance of cyanobacteria, indicating that CYRG can improve the intestinal microbial environment of broilers and protect intestinal health.

When intestinal diseases and inflammation occurs, they can cause an imbalance of intestinal flora. Most members of the Firmicutes are beneficial bacteria, such as Lactobacillus and Fecal bacilli. They produce acetic acid and butyric acid in the intestines, which helps promote the growth of intestinal epithelial cells and prevent pathogens from interfering with intestinal health (25). In this study, the results showed that CYRG could regulated the intestinal floral structure, which may be facilitated by increasing the relative abundance of intestinal probiotics (Firmicutes), and decreasing the numbers of harmful bacteria such as Proteobacteria and Cyanobacteria. Thus, CYRG affects the structure of intestinal microbiota, regulates the intestinal microecological balance, while promoting the health of broilers. Meanwhile, correlation analysis revealed that beneficial bacteria in the intestine (including *Lactobacillus salivarius* and *Lactobacillus crispatus*) were positively associated with intestinal IgA and IgG. Moreover, *Ruminococcaceae bacterium* were positively correlated with CAT and GSH-Px activities, and *Unclassified Clostridia ucG 014* was negatively correlated with MDA. Thus, CYRG may improve intestinal function, enhance immunity and maintain intestinal health by adjusting the intestinal flora.

Therefore, CYRG supplementation was beneficial to growth performance, slaughter performance, immunity and antioxidant capacity of broilers. At the same time, CYRG can regulate the cecal floral structure in broilers, increase the abundance of beneficial bacteria and decrease the quantity of harmful bacteria, reduce the risk of intestinal inflammation and maintain the intestinal barrier. CYRG has potential application value in promoting the growth of broiler chickens and preventing intestinal inflammatory diseases.

It’s generally considered that traditional Chinese medicines are characterized by “multi-components” and “multi-targets.” Therefore, the growth performance, body size trait, relative organ weight, slaughter performance, cytokines and antioxidant indicators in serum were detected in this paper. However, traditional Chinese medicine contains numerous ineffective and unknown ingredients (53–55), which brings about great difficulty in clarifying its application effect and in accurately applying it in actual production. So, the results in this study indicated that CYRG is effective in improving slaughter performance of broilers, which provides an objective data for the application of CYRG in poultry breeding. Nevertheless, there are still some shortcomings in this research. For example, if the feeding experiment could be conducted for a longer period of time, would it be possible to have a positive impact on the body size and growth performance of the broilers? This will be explored in more depth in future research, to ensure the assessment of the application potential of *Chinese yam*–*Rehmannia glutinosa* ‘medicine pair’ in the livestock industry more objective and impartial.

## 5 Conclusion

The dietary addition of *Chinese yam*–*Rehmannia glutinosa* ‘medicine pair’ had positive effects on slaughter performance, serum antioxidant capacity, immune function, and intestinal microecological balance of broilers. In addition, compared with supplementation of 1, 1.5, and 2% CYRG in the diet, the effect of supplementation 0.5% CYRG is more prominent. These results indicate that dietary 0.5% CYRG can improve the economic benefits of broilers by improving growth performance, slaughter performance, immune function, antioxidant capacity, and gut microbiota.

## Data Availability

The original contributions presented in the study are included in the article/supplementary material, further inquiries can be directed to the corresponding author/s.

## References

[1] Epping J Laibach N. An underutilized orphan tuber crop-*Chinese yam*: a review. Planta. (2020) 252:58. doi: 10.1007/s00425-020-03458-3, 32959173 PMC7505826

[2] Yu YG Guo XY Li XY Dai DD Xu XR Ge XJ . Organ- and age-specific differences of *Dioscorea polystachya* compounds measured by UPLC-QTOF/MS. Chem Biodivers. (2021) 18:e2000856. doi: 10.1002/cbdv.202000856, 33295037

[3] Zhang J Jin Y Cao M Deng J Chang Y Shi M . Effects of dietary *Chinese yam* polysaccharide copper complex on growth performance, immunity, and antioxidant capacity of broilers. Front Vet Sci. (2023) 10:1123002. doi: 10.3389/fvets.2023.1123002, 36875994 PMC9978188

[4] Wei C Lv S Jiang L Fang F Liu L Sun S . Yam polysaccharide extraction, purification, structural features, and biological properties: a review. Carbohydr Res. (2026) 559:109746. doi: 10.1016/j.carres.2025.109746, 41274220

[5] Price EJ Bhattacharjee R Lopez-Montes A Fraser PD. Metabolite profiling of *yam* (*Dioscorea* spp.) accessions for use in crop improvement programmes. Metabolomics. (2017) 13:144. doi: 10.1007/s11306-017-1279-7, 29104519 PMC5641283

[6] Guo L Chang Y Sun Z Deng J Jin Y Shi M . Effects of *Chinese yam* polysaccharide on intramuscular fat and fatty acid composition in breast and thigh muscles of broilers. Foods. (2023) 12:1479. doi: 10.3390/foods12071479, 37048300 PMC10094610

[7] Chang Y Zhang J Jin Y Deng J Shi M Miao Z. Effects of dietary supplementation of *Chinese yam* polysaccharide on carcass composition, meat quality, and antioxidant capacity in broilers. Animals (Basel). (2023) 13:503. doi: 10.3390/ani13030503, 36766389 PMC9913201

[8] Wang Y Liu Y Zhang Y Huo Z Wang G He Y . Effects of the polysaccharides extracted from *Chinese yam* (*Dioscorea opposita Thunb*.) on cancer-related fatigue in mice. Food Funct. (2021) 12:10602–14. doi: 10.1039/d1fo00375e, 34585194

[9] Deng J Zhang J Chang Y Wang S Shi M Miao G. Effects of *Chinese yam* polysaccharides on the immune function and serum biochemical indexes of broilers. Front Vet Sci. (2022) 9:1013888. doi: 10.3389/fvets.2022.1013888, 36148469 PMC9485930

[10] Meng X Hu W Wu S Zhu Z Lu R Yang G . *Chinese yam* peel enhances the immunity of the common carp (*Cyprinus carpio* L.) by improving the gut defence barrier and modulating the intestinal microflora. Fish Shellfish Immunol. (2019) 95:528–37. doi: 10.1016/j.fsi.2019.10.066, 31678187

[11] Jia J Chen J Wang G Li M Zheng Q Li D. Progress of research into the pharmacological effect and clinical application of the traditional Chinese medicine *Rehmanniae Radix*. Biomed Pharmacother. (2023) 168:115809. doi: 10.1016/j.biopha.2023.115809, 37907043

[12] Yang B Li X Badran AMM Abdel-Moneim AE. Effects of dietary incorporation of *Radix rehmanniae praeparata* polysaccharide on growth performance, digestive physiology, blood metabolites, meat quality, and tibia characteristics in broiler chickens. Poult Sci. (2023) 102:103150. doi: 10.1016/j.psj.2023.103150, 37871491 PMC10618489

[13] Yu L Lin F Yu Y Deng X Shi X Lu X . *Rehmannia glutinosa* polysaccharides enhance intestinal immunity of mice through regulating the microbiota. Int J Biol Macromol. (2024) 283:137878. doi: 10.1016/j.ijbiomac.2024.137878, 39571844

[14] Quan Y Jia F Hao H Nie Y Xu D Kang S . *Rehmannia glutinosa* libosch ameliorates diabetic nephropathy in Sprague-Dawley rats by the TLR4/MyD88/NF-κB signalling pathway. Fitoterapia. (2025) 184:106595. doi: 10.1016/j.fitote.2025.106595, 40334822

[15] NRC. Nutrient requirements of poultry. 9th ed. Washington, DC, USA: National Academic Press (1994).

[16] Ministry of Agriculture and Rural Affairs of the People’s Republic of China. Performance Ferms and measurements for poultry (NY/T823-2020). Beijing, China: China Agriculture Press (2020).

[17] Guo Y Zhang JL Xi L Wu J Han JC Yang GL . Oxidative stress mediated immunosuppression caused by ammonia gas via antioxidant/oxidant imbalance in broilers. Br Poult Sci. (2023) 64:36–46. doi: 10.1080/00071668.2022.2122025 36083210

[18] Li W Wei F Xu B Sun Q Deng W Ma H . Effect of stocking density and alpha-lipoic acid on the growth performance, physiological and oxidative stress and immune response of broilers. Asian Australas J Anim Sci. (2019) 32:1914–22. doi: 10.5713/ajas.18.0939, 31010966 PMC6819680

[19] Nasr M Alkhedaide AQ Ramadan A Hafez A Hussein MA. Potential impact of stocking density on growth, carcass traits, indicators of biochemical and oxidative stress and meat quality of different broiler breeds. Poult Sci. (2021) 100:101442. doi: 10.1016/j.psj.2021.101442, 34607150 PMC8493580

[20] Son J Kim HJ Hong EC Kang HK. Effects of stocking density on growth performance, antioxidant status, and meat quality of finisher broiler chickens under high temperature. Antioxidants (Basel). (2022) 11:871. doi: 10.3390/antiox11050871, 35624735 PMC9138006

[21] Liu Y Zhang Y Bai D Li Y He X Ito K . Dietary supplementation with chlorogenic acid enhances antioxidant capacity, which promotes growth, jejunum barrier function, and cecum microbiota in broilers under high stocking density stress. Animals (Basel). (2023) 13:303. doi: 10.3390/ani13020303, 36670842 PMC9854556

[22] Liu L Wang LP He S Ma Y. Immune homeostasis: effects of Chinese herbal formulae and herb-derived compounds on allergic asthma in different experimental models. Chin J Integr Med. (2018) 24:390–8. doi: 10.1007/s11655-018-2836-2, 29752613

[23] Kirrella AA Abdo SE El-Naggar K Soliman MM Aboelenin SM Dawood MAO . Use of corn silk meal in broiler diet: effect on growth performance, blood biochemistry, immunological responses, and growth-related gene expression. Animals. (2021) 11:1170. doi: 10.3390/ani11041170, 33921779 PMC8073180

[24] Pan XY Qiu ZY Liu C Wang C Wang X Huang LN . Effects of *Dioscorea oppositifolia* L. on growth performance, biochemical indicators, immunity, and intestinal health of weaned piglets. Front Vet Sci. (2025) 12:1529881. doi: 10.3389/fvets.2025.1529881, 40351768 PMC12063354

[25] Li X Gui R Wang X Ning E Zhang L Fan Y . Oligosaccharides isolated from *Rehmannia glutinosa* protect LPS-induced intestinal inflammation and barrier injury in mice. Front Nutr. (2023) 10:1139006. doi: 10.3389/fnut.2023.1139006, 36908905 PMC9996025

[26] Meng XL Liu XQ Ning CX Ma JN Zhang XY Su XJ . *Rehmanniae Radix* and *Rehmanniae Radix Praeparata* improve diabetes induced by high-fat diet coupled with streptozotocin in mice through AMPK-mediated NF-κB/NLRP3 signaling pathway. Zhongguo Zhong Yao Za Zhi. (2021) 46:5627–40. doi: 10.19540/j.cnki.cjcmm.20210323.302 34951216

[27] Deng J Zhang J Jin Y Chang Y Shi M Miao Z. Effects of *Chinese yam* polysaccharides on the muscle tissues development-related genes expression in breast and thigh muscle of broilers. Genes (Basel). (2022) 14:6. doi: 10.3390/genes14010006, 36672746 PMC9858316

[28] Chan YC Chang SC Liu SY Yang HL Hseu YC Liao JW. Beneficial effects of yam on carbon tetrachloride-induced hepatic fibrosis in rats. J Sci Food Agric. (2010) 90:161–7. doi: 10.1002/jsfa.3801, 20355026

[29] Li P Jing Y Qiu X Xiao H Zheng Y Wu L. Structural characterization and immunomodulatory activity of a polysaccharide from *Dioscotea opposita*. Int J Biol Macromol. (2024) 265:130734. doi: 10.1016/j.ijbiomac.2024.130734, 38462105

[30] Lv Z Fan H Zhang B Xing K Guo Y. Dietary genistein supplementation for breeders and their offspring improves the growth performance and immune function of broilers. Sci Rep. (2018) 8:5161. doi: 10.1038/s41598-018-23530-z, 29581465 PMC5979951

[31] Arif M Baty RS Althubaiti EH Ijaz MT Fayyaz M Shafi ME . The impact of betaine supplementation in quail diet on growth performance, blood chemistry, and carcass traits. Saudi J Biol Sci. (2022) 29:1604–10. doi: 10.1016/j.sjbs.2021.11.002, 35280529 PMC8913552

[32] Qu Q Ma Y Huang Y Gao X Xuan Z Chen X . Effects of *Chinese yam* peels on immunity and gut microbiota in cyclophosphamide-induced chickens and optimization of extraction process. Poult Sci. (2025) 104:106028. doi: 10.1016/j.psj.2025.106028, 41237582 PMC12663624

[33] Zhang C Shu Y Li Y Wang F Gan J Wang Y . *Chinese yam* (*Dioscorea*) polysaccharide ameliorates ulcerative colitis in mice via modulating disorders of intestinal microecology and metabolism. Int J Biol Macromol. (2025) 315:144110. doi: 10.1016/j.ijbiomac.2025.144110, 40360104

[34] Qiao H Ren H Liu Q Jiang Y Wang Q Zhang H . Anti-inflammatory effects of *Rehmannia glutinosa* polysaccharide on LPS-induced acute liver injury in mice and related underlying mechanisms. J Ethnopharmacol. (2025) 351:120099. doi: 10.1016/j.jep.2025.120099, 40484254

[35] Zhong T He J Zhao H Tan C Zhou W Wu C . *Oxalis corniculata* L. as a source of natural antioxidants: phytochemistry, bioactivities, and application potential. Antioxidants (Basel). (2025) 14:1352. doi: 10.3390/antiox14111352, 41300509 PMC12649735

[36] Huang YC Huang SJ Yang HY Tsai CY Chang HC Chi HC . Development of a compound herbal formulation (HBK) with antitumor and antioxidant functions for cancer adjuvant therapy. Phytomedicine. (2025) 147:157212. doi: 10.1016/j.phymed.2025.157212, 40934759

[37] Chen M Liu Y Bao X Yue Y Tong B Yang X . Potential of *Chinese yam* (*Dioscorea polystachya turczaninow*) by-product as a feed additive in largemouth bass (*Micropterus salmoides*): turning waste into valuable resources. Aquac Nutr. (2023) 2023:9983499. doi: 10.1155/2023/9983499, 37234450 PMC10208758

[38] Xu Y Yang H Zhang L Su Y Shi D Xiao H . High-throughput sequencing technology to reveal the composition and function of cecal microbiota in Dagu chicken. BMC Microbiol. (2016) 16:259. doi: 10.1186/s12866-016-0877-2, 27814685 PMC5097418

[39] Qin C Gong L Zhang X Wang Y Wang Y Wang B . Effect of Saccharomyces boulardii and *Bacillus subtilis* B10 on gut microbiota modulation in broilers. Anim Nutr. (2018) 4:358–66. doi: 10.1016/j.aninu.2018.03.004, 30564755 PMC6284224

[40] Huang Y Lv H Song Y Sun C Zhang Z Chen S. Community composition of cecal microbiota in commercial yellow broilers with high and low feed efficiencies. Poult Sci. (2021) 100:100996. doi: 10.1016/j.psj.2021.01.019, 33667869 PMC7937748

[41] Yi L Zhang Z Li Z Li Q Yang M Huang Y . Effects of citrus pulp on the composition and diversity of broiler cecal microbes. Poult Sci. (2023) 102:102454. doi: 10.1016/j.psj.2022.102454, 36682129 PMC10014344

[42] Hu X Zhen W Bai D Zhong J Zhang R Zhang H . Effects of dietary chlorogenic acid on cecal microbiota and metabolites in broilers during lipopolysaccharide-induced immune stress. Front Microbiol. (2024) 15:1347053. doi: 10.3389/fmicb.2024.1347053, 38525083 PMC10957784

[43] Zhang J Chai L Guo Y Han J Yang G. Dietary low levels of *Eucommia ulmoides* leaf extracts: effects on antioxidant capacity, immunity, and cecal microbiota in lipopolysaccharide-challenged broilers. Front Microbiol. (2025) 16:1662502. doi: 10.3389/fmicb.2025.1662502, 41113657 PMC12531263

[44] Chen F Zhang H Zhao N Yang X Du E Huang S . Effect of chlorogenic acid on intestinal inflammation, antioxidant status, and microbial community of young hens challenged with acute heat stress. Anim Sci J. (2021) 92:e13619. doi: 10.1111/asj.13619, 34409681

[45] Orso C Stefanello TB Franceschi CH Mann MB Varela APM Castro IMS . Changes in the ceca microbiota of broilers vaccinated for coccidiosis or supplemented with salinomycin. Poult Sci. (2021) 100:100969. doi: 10.1016/j.psj.2020.12.066, 33684651 PMC7938242

[46] Xiao Y Xiang Y Zhou W Chen J Li K Yang H. Microbial community mapping in intestinal tract of broiler chicken. Poult Sci. (2017) 96:1387–93. doi: 10.3382/ps/pew372, 28339527

[47] El Kaoutari A Armougom F Gordon JI Raoult D Henrissat B. The abundance and variety of carbohydrate-active enzymes in the human gut microbiota. Nat Rev Microbiol. (2013) 11:497–504. doi: 10.1038/nrmicro3050, 23748339

[48] Berry D. The emerging view of Firmicutes as key fibre degraders in the human gut. Environ Microbiol. (2016) 18:2081–3. doi: 10.1111/1462-2920.13225, 26842002

[49] Turnbaugh PJ Ley RE Mahowald MA Magrini V Mardis ER Gordon JI. An obesity-associated gut microbiome with increased capacity for energy harvest. Nature. (2006) 444:1027–31. doi: 10.1038/nature05414, 17183312

[50] Reid NM Addison SL Macdonald LJ Lloyd-Jones G. Biodiversity of active and inactive bacteria in the gut flora of wood-feeding huhu beetle larvae (Prionoplus reticularis). Appl Environ Microbiol. (2011) 77:7000–6. doi: 10.1128/AEM.05609-11, 21841025 PMC3187079

[51] Chen H Yan H Xiu Y Jiang L Zhang J Chen G . Seasonal dynamics in bacterial communities of closed-cage broiler houses. Front Vet Sci. (2022) 9:1019005. doi: 10.3389/fvets.2022.1019005, 36406086 PMC9669973

[52] Wu JX Huang H Yang L Zhang XF Zhang SS Liu HH . Gastrointestinal toxicity induced by microcystins. World J Clin Cases. (2018) 6:344–54. doi: 10.12998/wjcc.v6.i10.344, 30283797 PMC6163130

[53] Deng YQ Gao M Lu D Liu QP Zhang RJ Ye J . Compound-composed Chinese medicine of Huachansu triggers apoptosis of gastric cancer cells through increase of reactive oxygen species levels and suppression of proteasome activities. Phytomedicine. (2024) 123:155169. doi: 10.1016/j.phymed.2023.155169, 37992493

[54] Feng L Zhang M Gu J Wu C Jia X. Innovation and practice of ‘component structure’ theory of the material basis of traditional Chinese medicine. Zhongguo Zhong Yao Za Zhi. (2013) 38:3603–7. 24494539

[55] Zhang Q Li R Peng W Zhang M Liu J Wei S . Identification of the active constituents and significant pathways of guizhishaoyao-zhimu decoction for the treatment of diabetes mellitus based on molecular docking and network pharmacology. Comb Chem High Throughput Screen. (2019) 22:584–98. doi: 10.2174/1386207322666191022101613, 31642770

